# Health and mass unemployment events—developing a framework for preparedness and response

**DOI:** 10.1093/pubmed/fdy174

**Published:** 2018-10-05

**Authors:** A R Davies, L Homolova, C N B Grey, M A Bellis

**Affiliations:** Policy, Research and International Development Directorate, Public Health Wales, Cardiff, UK

**Keywords:** emergency planning, public health, social determinants

## Abstract

**Background:**

Mass unemployment events are not uncommon yet the impact on health is not well recognised. There is a need for a preparedness and response framework, as exists for other events that threaten population health.

**Methods:**

Framework informed by a narrative review of the impact of mass unemployment on health (studies published in English from 1990 to 2016), and qualitative data from 23 semi-structured interviews with individuals connected to historical national and international events, addressing gaps in published literature on lessons learnt from past responses.

**Results:**

Economic and employment shock triggered by mass unemployment events have a detrimental impact on workers, families and communities. We present a public health informed response framework which includes (i) identify areas at risk, (ii) develop an early warning system, (iii) mobilise multi-sector action including health and community, (iv) provision of support across employment, finance and health (v) proportionate to need, (vi) extend support to family members and (vii) communities and (viii) evaluate and learn.

**Conclusion:**

Mass unemployment events have an adverse impact on the health, financial and social circumstances of workers, families, and communities. This is the first framework for action to mitigate and address the detrimental impact of mass unemployment events on population health.

## Introduction

Major changes in trade and labour markets because of globalisation and financial crises can result in the loss of a large employer in a localised area. The economic impact of such mass unemployment events (MUEs) can be severe, but the cost to health and social inequalities can be even greater and endure across generations.^1^ The impact of the coalfield closures in the UK in the 1970/80s, for example, remains evident today with higher levels of mortality and morbidity^2,3^ and widening inequalities in affected areas.^4^

During the global recession from 2007–16, the European Globalisation Adjustment Fund provided €600 M (with an additional €427 M match-funded by Member States) to support local responses to 146 significant unemployment events.^5^ Longitudinal studies have demonstrated that MUEs and recession pose risks to health, and that impacts are greatest where the underlying social, health and economic policy is not protective or supportive.^6^

National and international sustainable health development policies recognise the importance of both employment, and supporting resilience to external shocks to achieving good health.^7–10^ However, while emergency planning frameworks exist for other external shocks that have a detrimental impact on health, such as flooding, a framework to inform response to reduce the impact of MUEs on population health is lacking.

This study aimed to address that gap, by reviewing international literature and drawing on national and international expertise to describe the impact of MUEs on populations, developing a public health informed response to inform future action.

## Methods

### Literature review

A narrative review of academic and grey literature was undertaken using systematic methods.^11^ A structured search strategy (Table 1) identified literature published in English from January 1990 to September 2016, from the following indexed databases: AMED, BMI, CINAHL, Cochrane Library, CRD Databases, EMBASE, HMIC, Library Catalogue and Knowledge Base (Soutron), Medline, NHS Evidence, NICE, PsychINFO, Public Health Wales document database; and topic specific databases ASSIA, Econlit, Emerald Insight, Social care online, Sociological abstracts, Scopus. After duplicates were removed, the search yielded 69 citations. Grey literature (including government and policy think tanks websites and a structured Google search) yielded a further 104 citations.

**Table 1:** Key search terms and MESH subject headings used in the search strategy

***Key search terms***
(Automotive or car) (manufactur* or industr*)
Coal min*; Factories (manufacturing or manufacturer$)
Steelwork*; Steel plant$
involuntary job loss*; job displacement
closure$ adj3(plant$ or factory or factories or mine$ or industr*)
Mass adj2 (unemployment or redundan* or closure$)
large scale (unemployment or redundanc*) (lay off$)
Redundan*; Retrenched worker$;
Communit*; Community resilience; Community support; Individua support
(approach* or response$); (mitigation or mitigating)
(recovery or sustainability); Regeneration;
Health impact
**Subject Headings: MESH**
Economic Recession; Personnel Downsizing; Unemployment;
Coal mining; Industry; Steel;
Depression; Health status; Public health/ec, sn, td; Stress, Psychological; Quality of Life; Residence characteristics;

A total of 173 articles and reports were retrieved. Study titles were screened and relevant articles retained and reviewed. Only papers referring to MUEs from the industrial/manufacturing sector, exploring response, or wider health and social impacts on individuals and communities were included. After screening for relevance, a total of 108 citations were included in the final review.

### Semi-structured interviews

Practical lessons for action are difficult to ascertain from published literature. Thus we completed semi-structured interviews with individuals with experience in responding to MUEs. Individuals were recruited by purposive/snowball sampling, including those named in documented events and identified by authors of published reports. Forty-four individuals were invited by email to participate. Twenty-three accepted (52% response rate) including policy-makers (*n* = 6), health professionals (*n* = 6) and academics (*n* = 11), drawing on 12 MUE events across eight countries (Table 2). Interview topic guides, informed by the review, included the health and social impact on individuals, families and communities, and prevention and response. Interviews were conducted between September and December 2016, of 60–90 min duration and delivered face to face (*n* = 5), by telephone (*n* = 10) or video conference (*n* = 8).

**Table 2:** Characteristics of the past mass unemployment events drawn on during qualitative interviews

Event	Country	Total job losses	Year(s)	Number of interviewees
Nokia and Microsoft R&D Unit, Salo	Finland	6000	2012–16	2
Saab Automobile AB, Trollhättan	Sweden	3064	2011	1
Mitsubishi, Tonsley Park, Adelaide	Australia	1200	2004/05	3
		1700	2008	
Solid Energy/Spring Creek Mine, Greymouth	New Zealand	360	2012	2
Brisling Sardine Factory, Hetlevik	Norway	100–150	1975	1
Anglesey Aluminium, Holyhead	UK	400	2009	1
		90	2013	
Tata Steel, Port Talbot	UK	750	2016	3
MG Rover, Longbridge, Birmingham	UK	6300	2000–05	1
Coal Mines, Abertillery, South Wales	UK	N/A	1985	1
British Petroleum, Llandarcy	UK	750	1985	1
		150	1992/93	
		227	1997–99	
Corus (Ebbw Vale plant)	UK	780	2001/02	6
Sydney Steel Plant, Cape Breton, Nova Scotia	Canada	800	2003	1

All interviewees gave their informed consent and all interviews were recorded, transcribed and analysed thematically using ATLAS.ti Version 7.1. [Computer software (2013) Berlin, Scientific Software Development]. After analysis participants were asked for feedback on the key themes.

## Results

### Impact on individuals, families and communities

#### Individuals

Key findings from quantitative studies demonstrate the detrimental impact of MUEs and job insecurity on health in the short and long-term (Table 3). Studies report increased: use of primary and secondary care services^1,12^; alcohol-related hospitalisation and death^13,14^; chronic ill-health^2,12^; increased excess mortality^1,14^; including from circulatory disease^2,12,15^; poor mental health^13,14,16^; self-harm and suicide^14^ and increased health harming behaviours.^13,14,17^ For example, a 6-year observational study, using the European Health and Retirement Survey, found job loss was associated with increased risk of hazardous drinking.^18^ Further, a UK controlled study showed increased health service use; long-term redundant workers consulted their general practitioners (GPs) 57% more often, and attended hospital twice as much compared to those who had found re-employment.^12^ The uptake of social support due to ill health has also been shown to increase immediately following a MUE.^16,19,20^

**Table 3:** Summary of risk of adverse health outcome (physical and mental health) following job loss as a result of a mass unemployment event

	Risk of adverse health outcome			
Health outcome (source)	After 1 year	After 4 years	Longer-term	
Reporting less than good health^2^			10–20 years after coalmine closures	OR 1.24 (1.12–1.37)
Long-term limiting illness^2^			10–20 years after coalmine closures	OR 1.39 (1.25, 1.55)
All-cause mortality^14^	HR 1.79 (1.42, 2.26)	HR 1.35 (1.21, 1.50)	20 years later	HR 1.11 (1.06, 1.17)
Mortality due to circulatory disease^14^	HR 2.28 (1.58, 3.30)	HR 1.55 (1.31, 1.85)	20 years later	HR 1.18 (1.09, 1.28)
Mortality due to alcohol-related disease^14^	HR 2.64 (1.04, 3.42)	HR 1.66 (1.13, 2.45)	15 years later	HR 1.27 (1.06, 1.53)
Admission to hospital for alcohol-related disease			12 years later^13^	Men: HR 1.22 (1.05, 1.41)
				Women: HR 1.43 (1.10, 1.87)
			20 years later^14^	HR 1.22 (1.11, 1.34)
Mortality due to suicide^14^	HR 3.13 (1.33, 7.33)	HR 1.62 (1.08, 2.43)		
Mortality due to mental ill health^14^	HR 4.48 (1.56, 12.85)			
Morbidity (hospitalisation) due to mental ill health^14^	HR 1.63 (1.29, 2.04)	HR 1.32 (1.17, 1.49)	20 years later^14^	HR 1.19 (1.11, 1.27)
			Admission to hospital for self-harm (8 years later) ^28^	RR 2.47 (1.04, 5.89)

HR, hazard ratio; RR, risk ratio; OR, odds ratio with (95% Confidence Intervals provided in brackets).

Summary of studies quantifying health impacts (including general health, long-term chronic conditions; admissions to hospital, mortality and morbidity risks) from job loss linked to a mass unemployment events, with reported increased risks for adverse health outcomes still evident after 1 year, 4 years, and even 10–20 years later. Figures presented are from studies of industrial plant closure, published after 2000. Most quantitative estimates are from large international cross-sectional studies, analysing retrospective routine data, which do not control for underlying health and health behaviour.

The impact on physical and mental health was a common theme amongst those interviewed:

‘It is not only re-employment but there are so many implications for health and social […circumstances] as well’ (Events: Nokia and Microsoft R&D Unit, Finland 2012–16).

#### Families

Evidence of the impact on families is largely from qualitative studies. Whilst families provide a strong source of support for workers facing redundancy,^21,22^ the strain on family relationships following a MUE can be significant. Qualitative studies report an increase in divorce, conflict and domestic violence, unwanted pregnancy, spouse and child health, and financial hardship affecting parenting, child mental health and educational attainment.^20,23,24^ Following the coalmines closures in Wales, wives of displaced workers were found to ‘suffer in silence’, supporting their spouse and keeping the family together, while not receiving support themselves.^25^ Following plant closures in Germany, spouses reported similar levels of psychological distress to those made redundant.^26^ Families make major changes to work and living patterns with a detrimental impact on education of children.^27^ Interviewees reflected on the impact on the family:

‘People will be extremely anxious and all those impacts on family life will be there.’ (Event: Corus, Wales, 2001/02).

In addition, the impact extends to children, described as:

‘…feeling stress because of stress in the family.’ (Events: Nokia and Microsoft R&D Unit, Finland 2012–16).

Studies suggest the impact of unemployment, job insecurity and lower earnings can extend across generations.^28,29^ Ecological cross-sectional studies amongst populations of old industrial areas have demonstrated job loss is associated with increased social support payments, extending to the next generation.^19,30^

#### Communities

Evidence from the literature review, largely qualitative studies and interviews suggest the impact on the wider community can be ‘economic’ due to direct and indirect (through supply chain) job losses,^17,31–34^ reductions in the labour market for years following closure^27^ and spending power that supports the community and ‘psychological’ (due to a loss of collective identity).

The loss of a large localised employer can result in the loss of social support networks, and decline in community participation, contributing to a sense of grief and social isolation.^17,35,36^ As reflected across events:

‘You get people talking about the heart ripped out of the community.’ (Event: Corus, Wales, 2001/02).

‘You stay at home and you isolate yourself from the other world and you think you are the only one unemployed.’ (Event: Nokia and Microsoft R&D Unit, Finland, 2012–16).

The loss of the cultural reference group, especially for skilled occupations, can have a detrimental impact on work, family and community relationships, self-image, sense of values and optimism for the future.^35^ As reflected by one interviewee:

‘The lack of community connectedness now and the lack of identity and actually, this is our history, but how do we look to the future because there is no future in mining.’ (Event: Solid Energy, New Zealand, 2012)

Interviewees emphasised the potential for widening inequalities, especially amongst existing long-term unemployed in the affected area ineligible for targeted redundancy support and facing increased employment competition. MUEs can exacerbate social inequalities where inability to travel further for work, or local property devaluation impacts affordability of relocating,^27,37^ are barriers to mobility for re-employment.

### Response framework

**Fig. 1:**
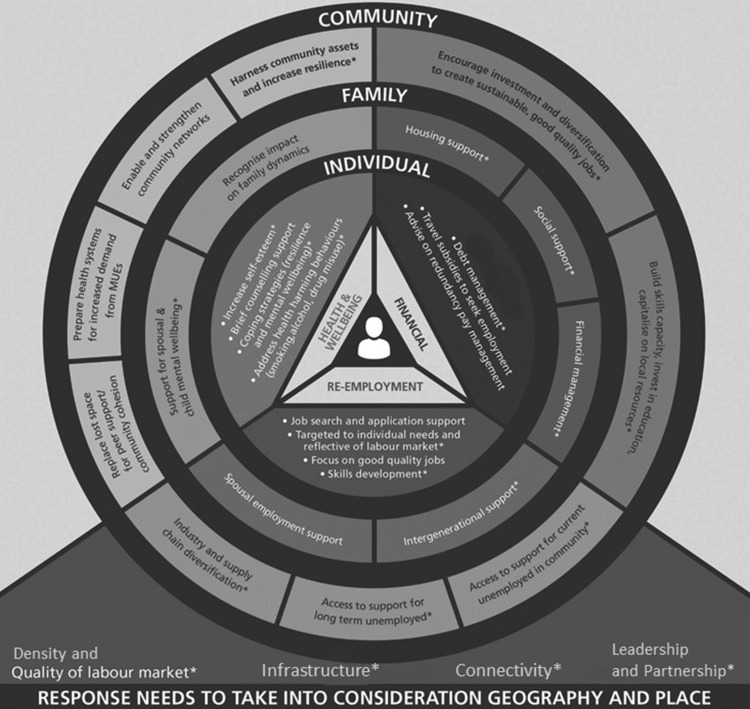
A response framework for Mass Unemployment Events: centred around three core elements addressing: the employment, financial, health and psychosocial support needs of workers, families and communities.

The response framework derived from the review and interviews addresses the re-employment, financial, health and wellbeing needs of individuals; extending support to family members and communities; and taking into account the local labour market, infrastructure, connectivity, and need for strong leadership and partnership working (Fig. 1). Eight key steps were identified for implementation:

#### (i) Identification of areas at risk

Proactive identification of industries at risk within the wider labour market context is needed to pre-empt response, and can be achieved through understanding economic forecasts,^38,39^ identification of ‘anchor’ companies with a significant presence in the local economy.^40^ The potential economic and social impact can be ascertained by the inclusion of health impact assessments and sharing evidence with strategic partners to mobilise collective action to address health earlier.^41–44^ The importance of building resilience amongst individuals and communities to better respond to events was also a common theme expressed by interviewees:

‘Let’s deliberately try to build the psychological assets of this community, well before they lose their job and well before they are told here is a voucher for vocational training.’ (Event: Mitsubishi Motors, Australia, 2004/05 & 2008)

#### (ii) Early warning

Early notification of the scale of the event (including number, skill mix, and geographical spread of workers affected), is crucial to inform timely mobilisation of resources and support.^38,45^ Liaising with employers to identify those at risk of redundancy to facilitate delivery and raise awareness of support available is essential. Interviewees reflected on the importance of addressing uncertainty quickly to prevent detrimental impacts on health:

‘…the anticipatory phase where people know they are losing their jobs or they think they are losing their jobs, they don’t know what they are going to do in their lives and they have no sense of control over their kind of lives.’ (Event: Corus, Wales, 2001/02)

#### (iii) Mobilise multi-sector response including health and community

Strong leadership alongside adopting an emergency planning approach ensures partnership working across key stakeholders and prompt access to support.^46,47^ Open and transparent communication alongside clear lines of reporting and governance are also critical for success.^48,49^ The inclusion of strategic input from health and community partners is essential to ensure the acute and longer-term health risks are understood and addressed^50^ and local psychosocial support is mobilised—which in some cases was more highly valued by those affected than governmental support.^51^ As described by one interviewee:

‘Health should be there in place from the start, and help to put the messages across in terms of managing the situation, around managing uncertainty.’ (Event: Tata Steel, Wales, 2016)

#### (iv) Advice and support addressing employment, financial advice and health

Responsive action focuses on securing re-employment for workers, career counselling and skills development are more effective when tailored to needs, delivered by high quality providers and relevant to the local labour market.^47,52^ However, job fairs,^21^ and support to start new businesses^53^ have variable uptake and success. Those that find re-employment more quickly tend to have higher overall job quality, lower anxiety, and higher life satisfaction.^21^ In line with Bourdieu’s concept of habitus, there are differences in adaptation to change predetermined by an individual’s social status, existing resources (capitals) and skillsets.^54^ As a result, some workers may be better positioned to utilise new opportunities, and find it easier to adapt.^54^

Securing good quality employment can reduce long-term health, social and economic consequences for workers, their households and wider community.^55^ Unsatisfactory jobs may lead to workers becoming ‘trapped’ in a precarious cycle of intermittent work and unemployment^27,56^ contributing to financial stress.^21,23^ Following loss of a car industry in Australia, one interviewee reflected on a rule of thirds:

‘About a third of the workers transitioned into reasonably secure employment, a third into less secure and a third really struggled, and were unemployed or under-employed.’ (Event: Mitsubishi, Australia, 2004/05 and 2008)

Provision of accessible financial advice and longevity of support were considered essential but at times overlooked:

‘There was quite a lot of softer kind of economic advice that could have been provided.’ (Event: Anglesey Aluminium, Wales, 2009 and 2013)

The financial strain of redundancy can also motivate seeking re-employment, whilst increasing anxiety with potential adverse consequences on securing re-employment.^57^ However, redundancy payment can also result in perceived financial security acting as a disincentive to act.^58^

Addressing health harming behaviours and psychosocial needs were considered key gaps in past events by those interviewed:^21,59^

‘It struck me very forcefully that nobody was addressing the longer-term impacts in terms of whether that be physical health or indeed mental health.’ (Event: Tata Steel, Wales, 2016)

Building the case for action to provide health and psychosocial support was a gap identified by many participants and supported by evidence from the literature.^51,60^ Suggested action includes evidencing the impact on health, identifying needs and sources of support within local communities, ensuring formal support addresses psychosocial needs^21,59,61^ and initiating proactive response across health services including preparedness for increased demand.

#### (v) Support proportionate to need

Those at risk of long-term unemployment identified by the participants and literature include older workers, unskilled workers and those less able to relocate for new employment.^17,25,52^ To ensure a population approach, these groups need targeted support potentially over a longer period of time, to prevent longer-term inequalities through limited access to the labour market, uptake of lower paid employment, resulting in withdrawal from the labour market.^1,19,21,30,49,53,57,62^

#### (vi) Extend support to family members

Interviewees reflected on the impact of the mass redundancy on family units and the need to extend support to family members. In some responses, provision included extending financial and debt management advice, re-employment support, health and wellbeing advice, including children; but the uptake was low and thought to be due to a lack of awareness.

#### (vii) Support the community and harness assets

The need to support the wider community, including those employed in the supply chain, was also highlighted:

‘Usually when we think of a redundancy or a plant closure it might just affect the workers, it doesn’t, it can affect the community; it can affect those people who supply parts to that company.’ (Event: British Petroleum, Wales, 1985)

For industrial MUEs, interviewees highlighted the need to recognise and address the impact of changes in a communities’ historical context and identity.^20^ Supporting communities to adapt to change by identifying and harnessing community assets, and building resilience.^17,25,27^

#### (viii) Evaluate the response

Interviewees recognised the difficulty and importance of examining the impact of the response extending beyond employment to health over the short- and longer-term, to help inform future action. Planning evaluation at the beginning of the response is possible.^63^

## Discussion

### Main findings of this study

This study sought to describe an evidence based framework for action to mitigate and address the detrimental impact of MUEs on population health. We have brought together evidence on the impact of mass unemployment on health at an individual, family and community level, alongside local and national perspectives experience from 23 individuals connected to 12 events across eight countries.

### What is already known on this topic?

Preparing and responding to MUEs is of relevance today, given the complex impact of globalisation and technological advances, alongside changing trade and economic environments, on employment. In 2018, the European Union unemployment level is expected to decline to 7.7%, the lowest level since 200 8^64^ but this trend masks a seven-fold variation in unemployment across the region from 3.5% in Czech Republic to 21.6% in Greece. In the UK, the Office for Budget Responsibility forecasts unemployment rate will remain around 5% to 2021, but comments that the impact of economic and policy uncertainties as the government negotiates leaving the European Union are unclear.^65^

The links between employment and achieving good health is recognised in national and international sustainable health development policies.^7–10^ A population approach, supporting individuals and communities, and in particular the most vulnerable, is needed to prevent widening health and social inequalities.

### What this study adds

Drawing on evidence from past events, we have developed a strategic preparedness and response framework to MUEs to address the impact across individuals, families and communities. The framework places emphasis on the societal impact and highlights the importance of addressing the health impact alongside re-employment. The potential for widening social inequalities through more detrimental impact amongst individuals and communities who are less skilled, older, less able to travel for better quality employment and less able to adapt to change is highlighted.^63^

Our findings reflect Bourdieu’s concept of habitus, where sudden changes in employment conditions can cause significant disruption to individual and community collective habitus (i.e. identity and values). Functioning in a new context with a potential change to social status requires adaptation to thrive.^54^ An individual’s personal skillsets, resources and capital can determine their sense of control over the situation and ability to adapt.^66^ An increase in precarious employment, where jobs for life are no longer guaranteed,^54^ means individuals need skills to adapt to change^1,67^ and some groups, particularly the older workforce^27,21,52^ may require more targeted support to thrive.

The impact may extend across generations, as a period of unemployment in young men under 23 years increases the likelihood of future unemployment and a cycle of no work/low wage across a life-course.^17^ Longer-term action to strengthen the labour market (large, small and medium enterprises), strengthen the local infrastructure are also essential to prevent such events and support sustainable recovery.^58,68,69^ Successful implementation of a comprehensive response to MUEs, addressing the potential health and wider social impact, requires strong leadership and partnership working across sectors.

### Limitations of this study

We sought to address gaps in the academic literature on practical lessons learnt through the inclusion of grey literature and interviews with academics and those involved in local, regional or national response. However, identifying individuals with knowledge of past responses was challenging, and the views of employers were not included. The evidence base was limited by the lack of published evaluations of past responses. Engagement with the wider public and community, alongside employers including public and private sector organisations would strengthen the framework by co-producing a response.

## Conclusions

Whilst governments along with public and private sector partners work to prevent such events, efforts are not always successful. Given the clear economic, social and health impact of MUEs, and current global, economic and political climates, this framework is an important tool to inform local and national coordinated action to minimise the consequences and harms of MUEs to population health. The application and implementation extends beyond the public health disciplines, to local and national partners across health, community and economy.
